# Genotype data for 60 SNP genetic markers associated with eye, hair, skin color, ABO blood group, sex, core Y-chromosome haplogroups in Kazakh population

**DOI:** 10.1186/s13104-024-06712-z

**Published:** 2024-02-18

**Authors:** Alizhan Bukayev, Baglan Aidarov, Denis Fesenko, Viktoriya Saidamarova, Ivan Ivanovsky, Elina Maltseva, Dinara Naizabayeva, Ayagoz Bukayeva, Bekzhan Faizov, Vladimir Pylev, Akynkali Darmenov, Yuriy Skiba, Elena Balanovska, Maxat Zhabagin

**Affiliations:** 1 National Center for Biotechnology, Astana, 010000 Republic of Kazakhstan; 2 Engelhardt Institute of Molecular Biology, Russian Academy of Sciences, Moscow, 119991 Russia; 3 Karaganda Academy of the Ministry of Internal Affairs of the Republic of Kazakhstan Named After Barimbek Beisenov, Karaganda, 100000 Republic of Kazakhstan; 4 DNA Research Center, LLC, Khimki, 141402 Russia; 5 Almaty Branch of the National Center for Biotechnology, Almaty, 050054 Kazakhstan; 6 Tethys Scientific Society, Almaty, 050063 Kazakhstan; 7 Bochkov Research Centre of Medical Genetics, Moscow, 115522 Russia

**Keywords:** Forensic DNA Phenotyping (FDA), SNP Genotyping, Microarray, Eye color, Hair color, Skin color, AB0 blood group, Y-chromosome haplogroup, Sex, Kazakh population

## Abstract

**Objectives:**

The collection of genotype data was conducted as an essential part of a pivotal research project with the goal of examining the genetic variability of skin, hair, and iris color among the Kazakh population. The data has practical application in the field of forensic DNA phenotyping (FDA). Due to the limited size of forensic databases from Central Asia (Kazakhstan), it is practically impossible to obtain an individual identification result based on forensic profiling of short tandem repeats (STRs). However, the pervasive use of the FDA necessitates validation of the currently employed set of genetic markers in a variety of global populations. No such data existed for the Kazakhs. The Phenotype Expert kit (DNA Research Center, LLC, Russia) was used for the first time in this study to collect data.

**Data description:**

The present study provides genotype data for a total of 60 SNP genetic markers, which were analyzed in a sample of 515 ethnic Kazakhs. The dataset comprises a total of 41 single nucleotide polymorphisms (SNPs) obtained from the HIrisPlex-S panel. Additionally, there are 4 SNPs specifically related to the AB0 gene, 1 marker associated with the AMELX/Y genes, and 14 SNPs corresponding to the primary haplogroups of the Y chromosome. The aforementioned data could prove valuable to researchers with an interest in investigating genetic variability and making predictions about phenotype based on eye color, hair color, skin color, AB0 blood group, gender, and biogeographic origin within the male lineage.

**Supplementary Information:**

The online version contains supplementary material available at 10.1186/s13104-024-06712-z.

## Objective

Genotype data was collected as part of a pivotal study to study the genetic variability of skin, hair, and iris color in the Kazakh population. The aim of the pivotal study is to evaluate the accuracy of predicting the color of the skin, hair, and iris using the genetic markers of the HIrisPlex-S set [1]. Genotype data has been published for the first time. This data will be integrated with phenotypic data for analysis in a forthcoming article, according to the purpose of the pivotal study.

The Kazakh population is classified as belonging to a mixed anthropological type known as the South Siberian race. This race can be identified as a transitional group that emerged from the intermingling of Mongoloid and Caucasoid populations in the southern regions of Siberia, Kazakhstan, and Central Asia [2]. The geographic range inhabited by the Kazakh population spans from the western banks of the Volga River to the eastern slopes of the Altai Mountains. It extends from the northern West Siberian lowland to the southern Kyzylkum desert and the Tien Shan mountain system. This vast territory ranks ninth globally in terms of its expanse. Hence, it is reasonable to anticipate a considerable range of phenotypic variations in facial characteristics within the Kazakh population. Nevertheless, it is worth noting that the phenotypic characteristics associated with light skin Kazakh individuals may possess unique genetic underpinnings in terms of pigmentation. This is due to the fact that communities residing in the border regions between Asia and Europe exhibit genetic variations that distinguish them from European populations [3].

The study has applications in several fields: medicine (pigmentation pathology) [4], physical anthropology and archeology (reconstruction of appearance from ancient DNA) [5, 6], as well as forensic DNA phenotyping (FDA) [7, 8]. Due to the limited size of forensic databases from Central Asia (Kazakhstan), it is practically impossible to obtain an individual identification result based on forensic profiling of short tandem repeats (STRs). Consequently, the latter field is particularly pertinent in situations where it is impossible to obtain an individual identification result. Therefore, the FDA is developing swiftly today, including the prediction of a person's externally visible characteristics in relation to their appearance, biogeographic origin, and age based on their DNA [9]. However, their widespread use in forensic practice requires validation of the proposed sets of genetic markers in different populations around the world. There was no such data for the Kazakhs. In this study, data was obtained for the first time using the Phenotype Expert kit (DNA Research Center, LLC, Russia) [10].

## Data description

Genotype data was obtained for 515 individuals from the Kazakh population. Individuals were selected from volunteers who took part in this study. Each individual familiarized himself with the aims and methods of the study, signed an informed consent for his/her participation, and provided 10 ml of venous blood for the study. The Local Ethical Commission at the National Center for Biotechnology (Kazakhstan) has previously reviewed and approved procedures relating to blood sampling and other research methods (No. 3 of August 7, 2020). The criteria for random selection included individuals who had resided in Kazakhstan for at least three generations, with the exclusion of cousins within three generations. The sample under study comprises 162 female participants and 353 male participants, with mean ages of 22 years for females and 21 years for males.

The isolation of DNA from venous blood was conducted using the Wizard (R) Genomic DNA Purification Kit (Promega, USA) in accordance with the protocol provided by the manufacturer. The concentration of DNA was measured using a NanoDrop One instrument (Thermo Fisher Scientific, USA) and a Quantus Fluorometer (Promega, USA) with the QuantiFluor(R) ONE dsDNA System kit (Promega, USA) following the instructions provided by the manufacturer. Genotyping of markers associated with eye color, hair color, skin color, AB0 blood group, basic Y-chromosome DNA haplogroup, and gender was carried out using a Phenotype Expert kit (DNA Research Center, LLC, Russia). A test included multiplex PCR, hybridization of the PCR product on a biochip, and genotype determination. Multiplex PCR was performed to amplify and fluorescently label 53 target fragments contained 60 genetic markers associated with eye color, hair color, skin color, AB0 blood group, basic Y-chromosome DNA haplogroup and sex. The detailed description of Phenotype Expert kit has been provided previously [10]. PCR was performed in 25 μl of the following composition: PCR buffer with HotTaqMulti polymerase, 4 units (Asfogen, Russia), 5 mM MgSO_4_, 0.2 mM of each of the dNTPs (Sibenzym, Russia), primer mixture, 200 pmol of Cy5-TCATTGGATCTCATTA universal primer, 5 ng of genomic DNA. Amplification was carried out in 0.2 ml PCR-tubes on a CFX96 thermocycler (Bio-Rad, USA) with the following conditions: 95 °C for 2 min and 50 cycles in the first stage (95 °C for 20 s, 65 °C for 30 s, 66 °C for 30 s, 69 °C for 40 s), then 40 cycles in the second stage (95 °C for 20 s, 56 °C for 30 s, 72 °C for 30 s). The 30-µl chamber of the biochip was filled with a mixture of the following composition: 7.5 µl formamide, 7.5 µl 20 × SSPE, 15 µl PCR product. After incubation (12 h, 37 °C) and washing (10 min in 1 × SSPE at room temperature), the biochips were washed with distilled water, dried with compressed air, placed in a portable analyzer “Picodetect” (OOO BIOCHIP-IMB, Moscow) and fluorescence was registered with an exposure of 0.5–2 s according to the manufacturer’s manual. Image analysis was performed using ImaGel Studio software (EIMB RAS, Moscow). The result of automatic processing of each biochip was a text file with the genotype of the sample. Results obtained for the AB0 blood group, basic Y-chromosome haplogroup, and gender were interpreted using the Phenotype Expert program (DNA Research Center, LLC). The raw genotype data for the 60 genetic markers in the 515 Kazakhs are shown in Additional file 1: Table S1 and Table 1 [11]. We have submitted the data to the National Center for Biotechnology Information Reference Assembly dbSNP repository. The data is publicly accessible in the SNP Submission Batch, which can be viewed via the following link: (https://www.ncbi.nlm.nih.gov/SNP/snp_viewBatch.cgi?sbid=1063555).

**Table 1:** Overview of data files/data sets

Label	Name of data file/data set	File types (file extension)	Data repository and identifier (DOI or accession number)
*Data file 1*	*Raw genotype data for Kazakh population*	*VCF(vfc.gz)*	*dbSNP (*https://identifiers.org/dbsnp:rs1042602*)* [11]
			*dbSNP (*https://identifiers.org/dbsnp:rs1393350*)* [11]
			*dbSNP (*https://identifiers.org/dbsnp:rs1126809*)* [11]
			*dbSNP (*https://identifiers.org/dbsnp:rs12821256*)* [11]
			*dbSNP (*https://identifiers.org/dbsnp:rs12896399*)* [11]
			*dbSNP (*https://identifiers.org/dbsnp:rs2402130*)* [11]
			*dbSNP (*https://identifiers.org/dbsnp:rs17128291*)* [11]
			*dbSNP (*https://identifiers.org/dbsnp:rs1545397*)* [11]
			*dbSNP (*https://identifiers.org/dbsnp:rs1800414*)* [11]
			*dbSNP (*https://identifiers.org/dbsnp:rs1800407*)* [11]
			*dbSNP (*https://identifiers.org/dbsnp:rs12441727*)* [11]
			*dbSNP (*https://identifiers.org/dbsnp:rs1470608*)* [11]
			*dbSNP (*https://identifiers.org/dbsnp:rs1129038*)* [11]
			*dbSNP (*https://identifiers.org/dbsnp:rs12913832*)* [11]
			*dbSNP (*https://identifiers.org/dbsnp:rs2238289*)* [11]
			*dbSNP (*https://identifiers.org/dbsnp:rs6497292*)* [11]
			*dbSNP (*https://identifiers.org/dbsnp:rs1667394*)* [11]
			*dbSNP (*https://identifiers.org/dbsnp:rs1426654*)* [11]
			*dbSNP (*https://identifiers.org/dbsnp:rs3114908*)* [11]
			*dbSNP (*https://identifiers.org/dbsnp:rs3212355*)* [11]
			*dbSNP (*https://identifiers.org/dbsnp:rs312262906*)* [11]
			*dbSNP (*https://identifiers.org/dbsnp:rs1805005*)* [11]
			*dbSNP (*https://identifiers.org/dbsnp:rs1805006*)* [11]
			*dbSNP (*https://identifiers.org/dbsnp:rs2228479*)* [11]
			*dbSNP (*https://identifiers.org/dbsnp:rs11547464*)* [11]
			*dbSNP (*https://identifiers.org/dbsnp:rs1805007*)* [11]
			*dbSNP (*https://identifiers.org/dbsnp:rs201326893*)* [11]
			*dbSNP (*https://identifiers.org/dbsnp:rs1110400*)* [11]
			*dbSNP (*https://identifiers.org/dbsnp:rs1805008*)* [11]
			*dbSNP (*https://identifiers.org/dbsnp:rs885479*)* [11]
			*dbSNP (*https://identifiers.org/dbsnp:rs1805009*)* [11]
			*dbSNP (*https://identifiers.org/dbsnp:rs8051733*)* [11]
			*dbSNP (*https://identifiers.org/dbsnp:rs6059655*)* [11]
			*dbSNP (*https://identifiers.org/dbsnp:rs6119471*)* [11]
			*dbSNP (*https://identifiers.org/dbsnp:rs2378249*)* [11]
			*dbSNP (*https://identifiers.org/dbsnp:rs16891982*)* [11]
			*dbSNP (*https://identifiers.org/dbsnp:rs28777*)* [11]
			*dbSNP (*https://identifiers.org/dbsnp:rs12203592*)* [11]
			*dbSNP (*https://identifiers.org/dbsnp:rs12203592*)* [11]
			*dbSNP (*https://identifiers.org/dbsnp:rs683*)* [11]
			*dbSNP (*https://identifiers.org/dbsnp:rs10756819*)* [11]
			*dbSNP (*https://identifiers.org/dbsnp:rs10756819*)* [11]
			*dbSNP (*https://identifiers.org/dbsnp:rs8176741*)* [11]
			*dbSNP (*https://identifiers.org/dbsnp:rs8176720*)* [11]
			*dbSNP (*https://identifiers.org/dbsnp:rs8176719*)* [11]
			*dbSNP (*https://identifiers.org/dbsnp:rs1717135119*)* [11]
			*dbSNP (*https://identifiers.org/dbsnp:rs35284970*)* [11]
			*dbSNP (*https://identifiers.org/dbsnp:rs34820858*)* [11]
			*dbSNP (*https://identifiers.org/dbsnp:rs9786534*)* [11]
			*dbSNP (*https://identifiers.org/dbsnp:rs2740980*)* [11]
			*dbSNP (*https://identifiers.org/dbsnp:rs2032607*)* [11]
			*dbSNP (*https://identifiers.org/dbsnp:rs563604826*)* [11]
			*dbSNP (*https://identifiers.org/dbsnp:rs202084622*)* [11]
			*dbSNP (*https://identifiers.org/dbsnp:rs9341278*)* [11]
			*dbSNP (*https://identifiers.org/dbsnp:rs2032678*)* [11]
			*dbSNP (*https://identifiers.org/dbsnp:rs2319818*)* [11]
			*dbSNP (*https://identifiers.org/dbsnp:rs17307398*)* [11]
			*dbSNP (*https://identifiers.org/dbsnp:rs576940616*)* [11]
			*dbSNP (*https://identifiers.org/dbsnp:rs2032623*)* [11]
			*dbSNP (*https://identifiers.org/dbsnp:rs9341308*)* [11]
			*dbSNP (*https://identifiers.org/dbsnp:rs13447352*)* [11]

## Limitations

- Compared to comprehensive genome-wide data, the genotypes given for 60 genetic markers may not show all of the genetic variation in eye color, hair color, skin color, ABO blood type, gender, and the main Y-chromosome haplogroups in the Kazakh population.
- The limited size of the study sample (N = 515 individuals) drawn from the Kazakh population (16 million) may provide limitations in accurately evaluating the association between the examined SNPs and variations in eye, hair, and skin color.

### Supplementary Information

**Additional file 1.** Genotype data for 60 genetic markers associated with eye, hair, skin color, ABO blood group, sex, core Y-chromosome haplogroups in Kazakh population

## Data Availability

The data described in this Data note can be freely and openly accessed on the National Center for Biotechnology Information Reference Assembly dbSNP repository under SNP Submission Batch [https://www.ncbi.nlm.nih.gov/SNP/snp_viewBatch.cgi?sbid=1063555]. Please see Table 1 and references [11] for details and links to the data.

## Abbreviations

DNA: Deoxyribonucleic acid
FDA: Forensic DNA Phenotyping
STR: Short Tandem Repeat
SNP: Single nucleotide polymorphisms
dNTPs: Deoxynucleotide triphosphates
PCR: Polymerase chain reaction
SSPE: Sodium Chloride-Sodium Phosphate–EDTA
